# Omicron: a chimera of two early SARS-CoV-2 lineages

**DOI:** 10.1038/s41392-022-00949-5

**Published:** 2022-03-17

**Authors:** Xiuliang Liu, Jiasheng Xiong, Zhong Sun, Jingjing Hu, Karuppiah Thilakavathy, Mingquan Chen, Qi Zhao, Yi Feng, Qingwu Jiang, Chenglong Xiong

**Affiliations:** 1 Department of Epidemiology, School of Public Health, Key Laboratory of Public Health Safety, Ministry of Education, Fudan University, 200032 Shanghai, China; 2 Department of Social Medicine, School of Public Health; Key Laboratory of Public Health Safety, Ministry of Education, Fudan University, 200032 Shanghai, China; 3 Department of Biomedical Science, Faculty of Medicine and Health Sciences, Universiti Putra Malaysia, Serdang, 43400 Malaysia; 4 Shanghai Pinnacles Medical Technology Co., Ltd, 200126 Shanghai, China; 5 Genetics and Regenerative Medicine Research Group, Faculty of Medicine and Health Sciences, Universiti Putra Malaysia, Serdang, 43400 Malaysia; 6 Department of Emergency, Huashan Hospital, Fudan University, 200040 Shanghai, China; 7 Department of Integrative Medicine and Neurobiology, School of Basic Medical Sciences; Institutes of Brain Science, Brain Science Collaborative Innovation Center, State Key Laboratory of Medical Neurobiology, Institute of Acupuncture and Moxibustion, Fudan Institutes of Integrative Medicine, Fudan University, 200032 Shanghai, China

**Keywords:** Infectious diseases, Vaccines

**Dear Editor**,

The outbreak of the COVID‐19 that occurred in late 2019 has posed a remarkable threat to public health around the world. It is known that SARS-CoV-2 is a genetically diverse group that mutates continuously, leading to the emergence of multiple variants.^1^ Potential variants of concern (VOCs), variants of interest (VOIs), or variants under monitoring (VUMs) are regularly assessed based on the risk posed to global public health.

Following the identification of a novel variant in South Africa on 24 November 2021, WHO designated Omicron (clade GRA, PANGO lineage B.1.1.529 and descendants BA.1 and BA.2) as the fifth SARS-CoV-2 VOC 2 days later due to its large number of variations.^2^ The emergence and rapid spread of the Omicron variant characterize the current global epidemiology of SARS-CoV-2, where the continued decline in the prevalence of the previous Delta and other variants is observed.^3^ Despite its prompt predominance, knowledge gaps remain in their origin and evolution, fueling worldwide interests and speculations. Here, we propose that the prototype Omicron variant B.1.1.529 may be derived from the recombination of two early PANGO lineages of SARS-CoV-2.

We retrieved a total of 4192 whole-length genomes of SARS-CoV-2 from the EpiCoV^TM^ database of Global Initiative on Sharing All Influenza Data (GISAID) and SARS-CoV-2 data (NCBI). These genome sequences belong to 1,263 PANGO lineages, including 29 lineages of VOCs, VOIs, VUMs, and formerly monitored variants (FMVs) according to WHO’s Tracking SARS-CoV-2 variants (https://www.who.int/en/activities/tracking-SARS-CoV-2-variants/, accessed December 18, 2021), and are those with the earliest collection times within each PANGO lineage (Supplementary Table S1, and S2). By assessing the extent of sequencing completion, 2609 whole-length genomes of SARS-CoV-2 were used for the first round rapid screen (Extended Data 1, Genome sequence matrix 1). Subsequently, the genomic sequences involved in all putative recombination events identified in the first round of screening were singled out for further validation (Extended Data 2, Genome sequence matrix 2). Taking SARS-CoV-2/human/USA/UT-UPHL-211211887190/2021 (Accession, OL920485) as the representative of early prototype Omicron variant B.1.1.529 for querying, recombination events were detected and verified by Recombination Detection Program (RDP) v4.101 and the SimPlot Program package.

We confirmed that at least one recombination event occurred in the origin and evolutionary history of the prototype Omicron variant of SARS-CoV-2. In this event, strains belonging to PANGO lineage BA.1, like SARS-CoV-2/human/USA/COR-21-434196/2021 (Accession, OL849989), provided the fundamental genome for VOC Omicron and served as its major parents. While strains like SARS-CoV-2/human/IRN/Ir-3/2019 (Accession, MW737421) belonging to PANGO lineage B.35, as the minor parents, hybridized the genomic fractions into the major genome at the position of 21593-23118 nt (Fig. 1a and d and Supplementary Fig. S1). This fraction encodes 144–505 amino acid residues of SARS-CoV-2’s spike protein (S). As a result of the recombination, VOC Omicron did derive the substitutions of N211I, L212V, V213R, R214E, deletion215P, deletion216E, R346K, S371L, S373P, S375F, K417N, N440K, G446S, S477N, T478K, E484A, Q493R, G496S, Q498R, N501Y, Y505H, from the minor parent of SARS-CoV-2/human/IRN/Ir-3/2019-like strains. Another substitution of G/D339D may come from a back mutation after recombination. All these substitutions locate in the NTD (N-terminal domain, residues 18–330) and RBD (receptor-binding domain, residues 331–528) of the S1 subunit of spike protein,^4^ especially the latter where up to 16 substitutions occurred. The consistency of amino acid residues encoded by Omicron and its minor parent in the corresponding fraction and the difference between it and the major parent proved at the level of amino acids that the recombination event may have happened (Supplementary Table S3).

**Fig. 1:**
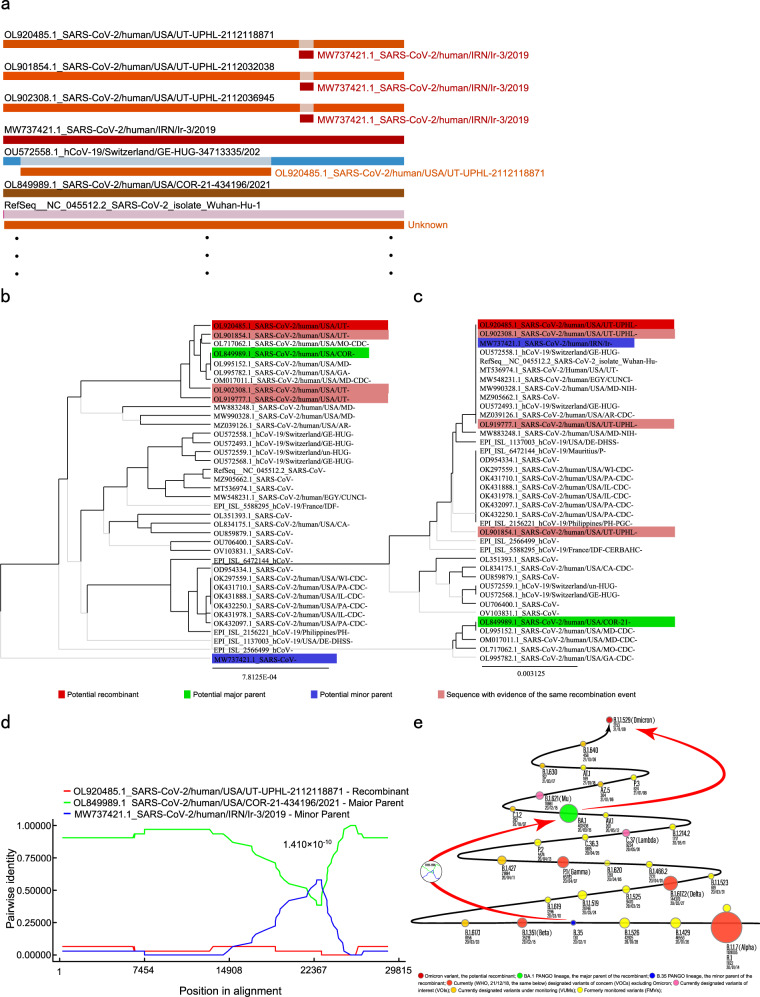
Panel of information related to the recombination event. **a** Schematic overview of the recombination events. Three representative isolates of prototype Omicron variant (PANGO lineage B.1.1.529), OL920485, OL901845, and OL902308, were hybridized into a genomic fraction from the minor parent MW737421 at the same position (21593-23118 nt). This recombination event can be detected via five statistical test methods, RDP (*P* = 1.410 × 10^−10^), GENECONV (*P* = 1.028 × 10^−8^), MaxChi (*P* = 8.468 × 10^−5^), Chimaera (*P* = 8.286 × 10^−5^), and 3Seq (*P* = 1.381 × 10^−7^). **b,** **c** Split UPGMA trees of the fractions derived from major and minor parents. The genetic distances between the prototype Omicron variant and the major and minor parents within the phylogenetic trees of non-recombined (**b**) and recombined (**c**) fractions are consistent with recombination signatures. In the non-recombination fraction, Omicron variants appear to be closer to the major parents, while in the recombination fraction, the opposite is true. **d** the plot diagram checked by the RDP method, shows its *P*-value. **e** The temporal order and isolation frequency of the PANGO lineages involves the recombination event and the VOCs, VOIs, VUMs, and FMVs. The dates that BA.1, B.35, the prototype Omicron variant, and other PANGO lineages were recorded into the database for the first time are displayed. The dot areas are calculated according to the natural logarithm of the isolate numbers within each PANGO lineage. B.35 is used as a reference to show the proportion of others. The data were derived from the EpiCoV^TM^ database of the Global Initiative on Sharing All Influenza Data (GISAID, accessed 23 January 2022). In **a, b, c**, and **d**, curves or sequences in red, green, and blue are potential recombinants, the major and minor parents, respectively.

It is known that the SARS-CoV-2 Omicron variant encodes 37 amino acid substitutions (including insertions and deletions) in the spike protein, and then the recombination event alone leads to 22 of them. Spike protein is the most crucial structural protein of SARS-CoVs. It plays a pivotal role in viral attachment, fusion, and entry and is also a target for developing antibodies, entry inhibitors, and vaccines. The recently emerged substitutions within the SARS-CoV-2 Omicron variant thereby raised concerns about the effectiveness of available vaccines and antibody therapeutics. It has been reported that these substitutions have led to some subtle variations in the spatial structure and the affinity to the hACE2 receptor of the spike protein.^5^ More importantly, it has caused the immune escape of the Omicron variant to the available vaccines and antibody therapeutics.^6^

According to the isolation frequency, BA.1 is a lineage with medium or high circulating intensity. There are 402,436 and 209,127 isolates in the GISAID’s EpiCoV^TM^ database and SARS-CoV-2 data of NCBI (both accessed January 23, 2022), accounting for 5.46% (402436/7373997) and 5.80% (209127/3599502) of the total isolates in the two databases, respectively, while B.35 is obviously a rare lineage, accounting for 0.0018% (130/7373997) and 0.0016% (58/3599502) respectively in the two databases, and its striking area is more limited to several countries, such as United Kingdom, Iceland, Australia, USA, Jordan, Timor-Leste, and New Zealand. Interestingly, both major parent BA.1 and minor parent B.35 are lineages that emerged at relatively early stages of the outbreak. The first records of them are 15 September 2020 and 11 February 2020, respectively (Fig. 1e). Considering the rapid transmission potential of lineage Omicron, which accounted for 71.9% of the isolating proportion in less than 2 months after the emergence, BA.1 could be the parent and not the descendant of VOC Omicron. It is the recombination that endows Omicron variants with the characteristic of rapid transmission. When investigating 5100 genomes of BA.1 PANGO lineage SARS-CoV-2, we found these viruses were different from the major parent of recombination only at 346 amino acid residue (R vs. K), in contrast to be fundamentally different from the index prototype Omicron variants (Accessions, OL920485, OL901845, and OL902308). It also indicated that BA.1 lineage should be the recombination parent of VOC Omicron rather than the descendant of it (Supplementary Table S3). However, the recombination event for VOC Omicron did not occur until recently. The reason may be that the circulating frequency of the lineage B.35 is too low, and its striking area is too limited, which reduces the chance of recombination between it and other lineages of SARS-CoV-2. After all, the prerequisite for recombination is that no less than two lineages of viruses co-infect an individual simultaneously, but the minor parent lineage B.35 is so rare that it hardly has the opportunity to infect an individual with a prior SARS-CoV-2 infection, and vice versa.

Our study suggested that chimerism is an important characteristic of the Omicron PANGO lineage SARS-CoV-2. It was generated by genomic recombination of two early SARS-CoV-2 lineages in the spike protein Coding Sequence (CDS). Recombination is proposed to be critical for coronavirus diversity and the emergence of SARS-CoV-2, MERS-CoV, SARS-CoV (2002), and other zoonotic CoVs. It allows viruses to overcome selective pressure and adapt to new hosts and environments. Viral recombination between different CoVs within animal populations may lead to the emergence of novel zoonotic CoVs that are lethal to humans. Studies comparing coronavirus strains closely related to SARS-CoV-2 had proposed that SARS-CoV-2 acquired the ability to infect human cells through recombination within the spike protein sequence.

Recombination may occur during infections in humans. The global spread and explosive growth of the SARS-CoV-2 in the human population have contributed additional mutational variability into this genome, increasing opportunities for future recombination. It has been reported that recombination among SARS-CoV-2 is associated with the increased spread and severe disease and has resulted in vaccine failure.^7^ Thus, targeting the ability of the virus to recombine is a critical consideration for vaccine development in the ongoing SARS-CoV-2 pandemic as well as future animal and zoonotic CoVs.

## Limitation

This study was carried out only based on bioinformatics analysis, therefore further laboratory analyses needed to confirm the in silico findings.

## Supplementary information

Supplementary Fig. S1

Table S1. Information of VOCs, VOIs, VUMs and FMVs of SARS-CoV-2 retrieval from GISAID

Table S2. Information of PANGO lineages of SARS-CoV-2 retrieval from NCBI

Table S3. Amino acid substitutions corresponding to the recombination fraction

Genome sequence matrix 1

Genome sequence matrix 2

## Data Availability

The data are available from the corresponding author on reasonable request, but GISAID data access, if needed, requires registration.
