# Supplementary material for: Omicron: a chimera of two early SARS-CoV-2 lineages
Source: Signal Transduct Target Ther. 2022 Mar 17;7:90. doi: 10.1038/s41392-022-00949-5 (PMC8927754; doi:10.1038/s41392-022-00949-5)
Supplement: Supplementary file 1 — Supplementary Fig. S1 [file 41392_2022_949_MOESM1_ESM.docx]

Supplementary Materials for

Omicron: a chimera of two early SARS-CoV-2 lineages

Xiuliang Liu, Jiasheng Xiong, Zhong Sun, Jingjing Hu, Karuppiah Thilakavathy, Mingquan Chen, Qi Zhao, Yi Feng, Qingwu Jiang, Chenglong Xiong

Correspondence to: [xiongchenglong@fudan.edu.cn](mailto:xiongchenglong@fudan.edu.cn), [fengyi17@fudan.edu.cn](mailto:fengyi17@fudan.edu.cn), [zhaoqi@shmu.edu.cn](mailto:zhaoqi@shmu.edu.cn)

**This PDF file includes:**

Materials and Methods

Figure. S1

Tables. S1 to S3

Captions for Data S1 to S2: Genome sequences used in this study

**Other Supplementary Materials for this manuscript include the following:**

Data S1 to S2

Data S1. Genome sequence matrix 1,

Data S2. Genome sequence matrix 2.

Materials and Methods

**Retrieval of complete SARS-CoV-2 genome sequences**

Complete SARS-CoV-2 genome sequences of 29 PANGO lineages of VOCs (variants of concern), VOIs (variants of interest), VUMs (variants under monitoring), and FMVs (formerly monitored variants) were downloaded from the EpiCoV^TM^ database of Global Initiative on Sharing All Influenza Data (GISAID) (<https://platform.epicov.org/>, accessed December 18, 2021). Considering the download speed of the servers and the flexibility for classifying according to PANGO lineages, genome sequences within other SARS-CoV-2 lineages were retrieved from the Nucleotide Records of SARS-CoV-2 data (NCBI) (<https://www.ncbi.nlm.nih.gov/sars-cov-2/>, accessed January 1, 2022). Three full-length genomes with the earliest collection time were downloaded in each PANGO lineage. Among them, there were 193 lineages, each with less than three genome sequences, and sequences within these lineages were all downloaded.

The genomes with poor sequencing quality were excluded and alternated according to the collection times. The criterion of poor quality is, i) more than 40 “n” (i.e., unknown bases) appeared continuously in a genome; or ii) the total number of “n” in a genome was more than 200.

**Sequence alignment**

Multiple sequence alignment for the genomes of SARS-CoV-2 was performed using MAFFT v7.407 (retree: 5; maxiter: 1000)^8,9^, and genome sequence SARS-CoV-2 isolate Wuhan-Hu-1 (Accession, NC045512) was used as the reference for trimming the head and tail of each genome.

**Recombination analysis**

Recombination Detection Program (RDP) version 4.101 was applied to detect recombination events in full-length genomes of SARS-CoV-2. Taking SARS-CoV-2/human/USA/UT-UPHL-211211887190/2021 (Accession, OL920485) as the query one, three statistical test methods of RDP, GENECONV, and MaxChi in RDP v4.101 were performed for the first round rapid determination. Putative recombination events with *P*< 0.01 (Bonferroni corrected) were identified and the genomes involved in all of these recombination events were picked out for further verification. In the second round of detection, seven methods of RDP, GENECONV, Bootscan, Maxchi, Chimaera, SiScan, and 3seq in RDP v4.101 package were performed with the same parameters (*P*< 0.01, Bonferroni corrected)^10,11^.

Split trees derived from major and minor parents were also inferred using the UPGMA method in RDP4 software^10,11^.

SimPlot 3.5.15 was used to determine the percent identity of the query sequence to reference sequences. Potential recombinant fractions among analyzed sequences were identified by sliding a 600-nt (nucleotides) window at a 10-nt step across the alignment using the Kimura two-parameter model^12,13^.

**Majority amino acid residues alignment for BA.1**

BA.1 PANGO lineage SARS-CoV-2 was downloaded on January 13, 2022, and 5,100 qualified genomes (refer to the above criterion of quality) remained for further sequence alignment. The unique genomic sequences were filtered out using CD-HIT^14^, requiring identity > 99.7%. The spike protein-coding sequence (CDS) was trimmed using the SARS-CoV-2 isolate Wuhan-Hu-1 as a reference. Protein translating and majority counting were performed by the MegAlign module of the Lasergene v7.1.0 software package^15^.

**Additional references:**

1. Haddad, D., *et al.* SARS-CoV-2: Possible recombination and emergence of potentially more virulent strains. *PLoS One* **16**, e251368 (2021).
2. Katoh, K. & Standley, D. M. MAFFT multiple sequence alignment software version 7: improvements in performance and usability. *Mol. Biol. Evol.* **30**, 772 (2013).
3. Martin, D. & Rybicki, E. RDP: detection of recombination amongst aligned sequences. *Bioinformatics* **16**, 562 (2000).
4. Martin, D. P., Murrell, B., Golden, M., Khoosal, A. & Muhire, B. RDP4: Detection and analysis of recombination patterns in virus genomes. *Virus Evol.* **1**, vev003 (2015).
5. Lole, K. S., *et al.* Full-Length Human Immunodeficiency Virus Type 1 Genomes from Subtype C-Infected Seroconverters in India, with Evidence of Intersubtype Recombination. *J. Virol.* **73**, 152 (1999).
6. Liu, J., *et al.* Characterization of SARS-CoV-2 worldwide transmission based on evolutionary dynamics and specific viral mutations in the spike protein. *Infect. Dis. Poverty* **10**, 112 (2021).
7. Li, W. & Godzik, A. Cd-hit: a fast program for clustering and comparing large sets of protein or nucleotide sequences. *Bioinformatics* **22**, 1658 (2006).
8. Anson, E. L. & Myers, E. W. ReAligner: a program for refining DNA sequence multi-alignments. *J. Comput. Biol.* **4**, 369 (1997).

.


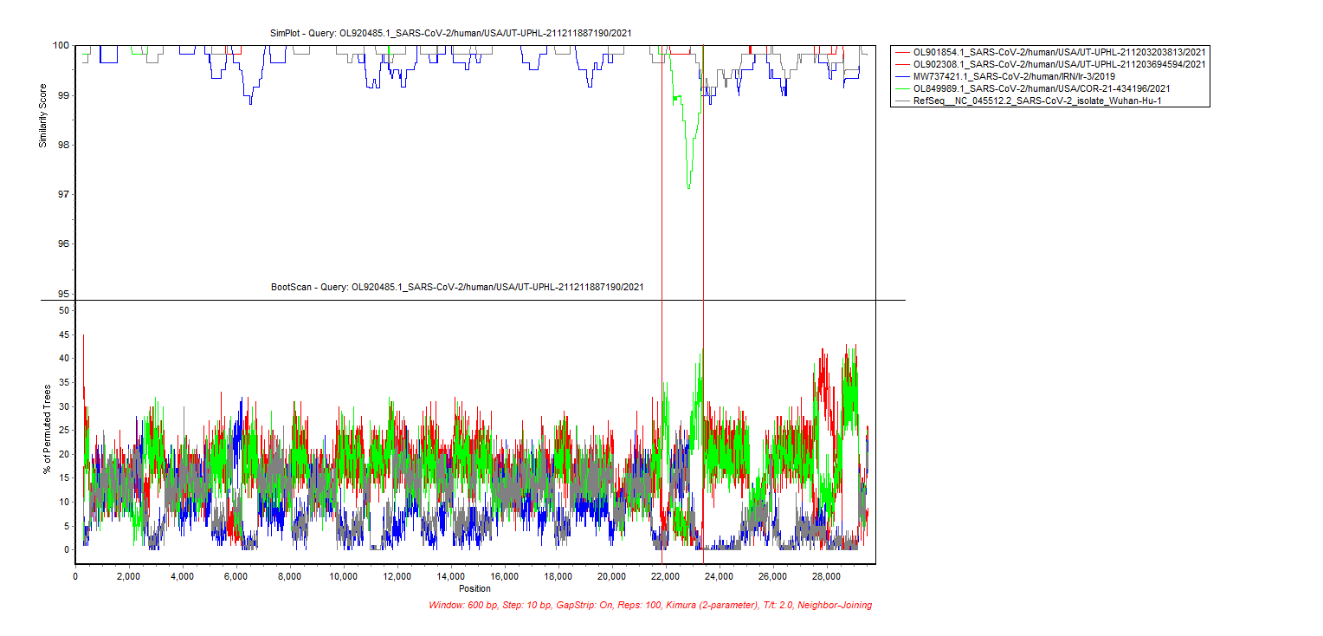
Figure S1. Recombination event verified by SimPlot 3.5.1 program package

The representative isolate of prototype Omicron variant (PANGO lineage B.1.1.529) OL920485 was hybridized into a genomic fraction from the minor parent MW737421 at the position 21593-23118 nt, and the other two isolates of Omicron OL901845 and OL902308 share the same characteristic of recombination with OL920485. The upper part shows the similarity among the genome sequence, and the lower part scans the recombination signal of them.

Table S1. Information of VOCs, VOIs, VUMs and FMVs of SARS-CoV-2 retrieval from GISAID

Table S2. Information of PANGO lineages of SARS-CoV-2 retrieval from NCBI

Table S3. Amino acid substitutions corresponding to the recombination fraction

Data S1. Genome sequence matrix 1 (rar. too large to upload as fasta format)

Data S2. Genome sequence matrix 2 (fasta.)
