# Supplementary material for: Omicron: a chimera of two early SARS-CoV-2 lineages
Source: Signal Transduct Target Ther. 2022 Mar 17;7:90. doi: 10.1038/s41392-022-00949-5 (PMC8927754; doi:10.1038/s41392-022-00949-5)
Supplement: Supplementary file 4 — Table S3. Amino acid substitutions corresponding to the recombination fraction [file 41392_2022_949_MOESM4_ESM.docx]

|  | 144 | 145 | 211 | 212 | 213 | 214 | 215* | 216* | 339 | 346 | 371 | 373 | 375 | 417 | 440 | 446 | 477 | 478 | 484 | 493 | 496 | 498 | 501 | 505 |
| --- | --- | --- | --- | --- | --- | --- | --- | --- | --- | --- | --- | --- | --- | --- | --- | --- | --- | --- | --- | --- | --- | --- | --- | --- |
| BA.1 majority | L | D | I | V | R | E | P | E | D | R | L | P | F | N | K | S | N | K | A | R | S | R | Y | H |
| **OL849989** | L | D | I | V | R | E | P | E | D | K | L | P | F | N | K | S | N | K | A | R | S | R | Y | H |
| **OL920485** | L | D | N | L | V | R | — | — | G | R | S | S | S | K | N | G | S | T | E | Q | G | Q | N | Y |
| **OL901845** | L | D | N | L | V | R | — | — | D | R | S | S | S | K | N | G | S | T | E | Q | G | Q | N | Y |
| **OL902308** | L | L | N | L | V | R | — | — | G | R | S | S | S | K | N | G | S | T | E | Q | G | Q | N | Y |
| **MW737421** | Y | Y | N | L | V | R | — | — | G | R | S | S | S | K | N | G | S | T | E | Q | G | Q | N | Y |
| NC_045512 | Y | Y | N | L | V | R | — | — | G | R | S | S | S | K | N | G | S | T | E | Q | G | Q | N | Y |

**Tab. S3 Amino acid substitutions corresponding to the recombination fraction***

*Note, Accession numbers in red, green, and blue are potential recombinants, the major and minor parents, respectively.
